# Determination of the Concentration of IgG against the Spike Receptor-Binding Domain That Predicts the Viral Neutralizing Activity of Convalescent Plasma and Serum against SARS-CoV-2

**DOI:** 10.3390/biology10030208

**Published:** 2021-03-10

**Authors:** Llipsy Santiago, Iratxe Uranga-Murillo, Maykel Arias, Andrés Manuel González-Ramírez, Javier Macías-León, Eduardo Moreo, Sergio Redrado, Ana García-García, Víctor Taleb, Erandi Lira-Navarrete, Ramón Hurtado-Guerrero, Nacho Aguilo, Maria del Mar Encabo-Berzosa, Sandra Hidalgo, Eva M. Galvez, Ariel Ramirez-Labrada, Diego de Miguel, Rafael Benito, Patricia Miranda, Antonio Fernández, José María Domingo, Laura Serrano, Cristina Yuste, Sergio Villanueva-Saz, José Ramón Paño-Pardo, Julián Pardo

**Affiliations:** 1 Biomedical Research Centre of Aragón (CIBA), Fundación Instituto de Investigación Sanitaria Aragón (IIS Aragón), 50009 Zaragoza, Spain; llipsysg@gmail.com (L.S.); iratxe.um@gmail.com (I.U.-M.); Sandra_Hidalgo_@hotmail.com (S.H.); diego_demiguel@hotmail.com (D.d.M.); rbenito@unizar.es (R.B.); 2 Instituto de Carboquímica (ICB), Consejo Superior de Investigaciones Científicas (CSIC), 50018 Zaragoza, Spain; maykelariascabrero@gmail.com (M.A.); sergio-redrado@hotmail.com (S.R.); eva@icb.csic.es (E.M.G.); 3 Institute for Biocomputation and Physics of Complex Systems (BIFI), Mariano Esquillor s/n, Campus Rio Ebro, Edificio I+D, 50018 Zaragoza, Spain; amgonram@gmail.com (A.M.G.-R.); jvmacleo@gmail.com (J.M.-L.); anaauxi.garcia@gmail.com (A.G.-G.); v.taleb.mail@gmail.com (V.T.); erandi@bifi.es (E.L.-N.); rhurtado@bifi.esC (R.H.-G.); 4 Department Microbiology, Preventive Medicine and Public Health, University of Zaragoza, 50009 Zaragoza, Spain; ejmoreo@gmail.com (E.M.); naguilo@unizar.es (N.A.); 5 Aragon I+D Foundation (ARAID), 50018 Zaragoza, Spain; 6 Laboratorio de Microscopías Avanzada (LMA), Mariano Esquillor s/n, Campus Rio Ebro, Edificio I+D, 50018 Zaragoza, Spain; 7 Copenhagen Center for Glycomics, Department of Cellular and Molecular Medicine, School of Dentistry, University of Copenhagen, DK-2200 Copenhagen, Denmark; 8 Biobanco de Aragón, Instituto Aragonés de Ciencias de la Salud (IACS), 50009 Zaragoza, Spain; mmencabo.iacs@aragon.es; 9 Unidad de Nanotoxicología e Inmunotoxicología (UNATI), Biomedical Research Centre of Aragón (CIBA), Fundación Instituto de Investigación Sanitaria Aragón (IIS Aragón), 50009 Zaragoza, Spain; aramirezlabrada@yahoo.es; 10 Servicio de Microbiología, Hospital Clinico Universitario Lozano Blesa, 50009 Zaragoza, Spain; 11 Banco de Sangre y Tejidos de Aragón, 50009 Zaragoza, Spain; pmirandica@hotmail.com (P.M.); jmdomingo1@hotmail.es (J.M.D.); 12 Department Animal Pathology, University of Zaragoza, 50013 Zaragoza, Spain; afmedica@unizar.es; 13 Servicio de Prevención de Riesgos Laborales, Hospital Clínico Universitario Lozano Blesa, 50009 Zaragoza, Spain; lserranoba@gmail.com (L.S.); cyuste@salud.aragon.es (C.Y.); 14 Department Pharmacology and Physiology, University of Zaragoza, 50013 Zaragoza, Spain; svs@unizar.es; 15 Servicio de Enfermedades Infecciosas, Hospital Clinico Universitario Lozano Blesa, 50009 Zaragoza, Spain; 16 Centro de Investigación Biomédicaen Red de Bioingeniería, Biomateriales y Nanomedicina (CIBER-BBN), 50018 Madrid, Spain

**Keywords:** coronavirus, SARS-CoV-2, IgG, antibody, convalescent plasma, ELISA

## Abstract

**Simple Summary:**

Passive immunization with hyperimmune plasma from convalescent patients has been proposed as a potentially useful treatment for COVID-19. Nevertheless, its efficacy in patients with COVID-19 remains uncertain. Thus, the establishment and validation of standardized methods that predict the viral neutralizing (VN) activity of plasma against SARS-CoV-2 is of utmost importance to appraise its therapeutic value. Using an in-house quantitative ELISA test and two independent cohorts with a total of 345 donors, we found that plasma and serum from most convalescent donors contained IgG antibodies specific to the spike receptor-binding domain (RBD) of SARS-CoV-2, with varying concentrations which correlate with previous disease severity and gender. Anti-RBD IgG plasma concentration significantly correlated with the plasma/serum VN activity against SARS-CoV-2 in vitro.

**Abstract:**

Several hundred millions of people have been diagnosed of coronavirus disease 2019 (COVID-19), causing millions of deaths and a high socioeconomic burden. SARS-CoV-2, the causative agent of COVID-19, induces both specific T- and B-cell responses, being antibodies against the virus detected a few days after infection. Passive immunization with hyperimmune plasma from convalescent patients has been proposed as a potentially useful treatment for COVID-19. Using an in-house quantitative ELISA test, we found that plasma from 177 convalescent donors contained IgG antibodies specific to the spike receptor-binding domain (RBD) of SARS-CoV-2, although at very different concentrations which correlated with previous disease severity and gender. Anti-RBD IgG plasma concentrations significantly correlated with the plasma viral neutralizing activity (VN) against SARS-CoV-2 in vitro. Similar results were found using an independent cohort of serum from 168 convalescent health workers. These results validate an in-house RBD IgG ELISA test in a large cohort of COVID-19 convalescent patients and indicate that plasma from all convalescent donors does not contain a high enough amount of anti-SARS-CoV-2-RBD neutralizing IgG to prevent SARS-CoV-2 infection in vitro. The use of quantitative anti-RBD IgG detection systems might help to predict the efficacy of the passive immunization using plasma from patients recovered from SARS-CoV-2.

## 1. Introduction

A new coronavirus responsible for severe acute respiratory syndrome (SARS), known as SARS-CoV-2, emerged in Wuhan, China in 2019 [1,2] and spread rapidly to the rest of the world. SARS-CoV-2 belongs to the family of human betacoronaviruses and it is the third member identified in this family causing a severe respiratory disease, behind the viruses SARS-CoV and Middle East respiratory syndrome (MERS) [3]. However, in contrast to SARS-CoV and MERS, which caused infections restricted to a limited number of countries, a few weeks after its identification SARS-CoV-2 had propagated all over the world, originating the pandemic known as coronavirus disease 19 (COVID-19). Despite the lethality of SARS-CoV-2 being significantly lower than that of SARS-CoV or MERS, the rapid propagation of the virus, having already infected millions of people, has caused more than half a million deaths and rising, a number far greater than that caused by SARS-CoV and MERS. A large proportion of COVID-19 patients are asymptomatic, which seems to facilitate viral spread. Among symptomatic patients, different stages of disease severity can be found ranging from mild (mild symptoms) to critical (viral sepsis symptoms including respiratory failure, shock, or multiorgan system dysfunction), with intermediate stages like moderate or severe pneumonia [4,5].

The immune response against SARS-CoV-2 is characterized by the activation of specific T- and B-cell responses against different viral epitopes, some of which are shared by SARS-CoV and seasonal coronaviruses [6,7,8,9,10,11]. In patients undergoing mild COVID-19 disease an efficient robust immune response characterized by the early activation of CD4+ T and cytotoxic CD8+ T cells seems to contribute to viral control and attenuation of disease progression [12,13]. In contrast, the early depletion of lymphocyte populations together with aberrant T cell hyperactivation, T cell exhaustion, and an exacerbated inflammatory response are the main features of the immune imbalance observed during severe COVID-19 [14,15].

Albeit huge efforts have been invested in developing effective treatments against COVID-19, most molecules with direct antiviral activity that have been clinically tested are showing limited efficacy [16]. Thus, the treatment of moderate, severe, and critical COVID-19 is mostly focused on supplemental oxygen and anti-inflammatory therapy to treat/prevent pneumonia, acute respiratory distress syndrome (ARDS), and cytokine release syndrome, the main events responsible for fatal outcomes [4,5].

As an alternative to synthetic and natural antiviral drugs, passive immunization has been used since early 20th century to treat different viral infections including SARS-CoV and MERS [17,18]. This old form of immunotherapy has emerged as one of the most promising treatments for severe/critical COVID-19. Thus, albeit the clinical evidence of efficacy and safety of convalescent plasma to treat COVID-19 was limited, due to the emergency situation, emergency use authorization (EUA) was granted by the FDA (Food and Drug Administration, US) to treat patients with severe disease and the first clinical trials suggested that it might be beneficial for patients with moderate-to-severe acute respiratory distress syndrome requiring a mechanical ventilation [19,20,21]. However, based on the most recent available evidence that does not support a clear role of convalescent plasma in patients with severe disease [22], the criteria has changed and EUA has been granted only for high-titer convalescent plasma among hospitalized patients with COVID-19 who are early in the disease course or have impaired humoral immunity.

Passive immunization consists of the transfer of pathogen-specific antibodies to patients whose immune system has not originated a response to control the infection [23]. In this way, donors’ antibodies help to neutralize and attenuate pathogen replication while the patient’s immune response activates to clear the infection. Among the different forms of passive antibody transfer is the use of plasma from convalescent patients who have recently recovered from an infection, which might be an effective and economic way to treat severe infections. However, the efficacy of this therapy in patients with COVID-19 remains controversial since both positive and negative results using convalescent plasma transfer have been reported in COVID-19 [19,20,24,25,26,27]. An apparent lack of standardization in the criteria used to select plasma with high neutralizing activity is one of the main problems that need to be overcome to optimize this treatment and improve its efficacy [23]. As an example of these limitations, a recent study found that the use of hyperimmune convalescent plasma was not beneficial for COVID-19 treatment. However, SARS-CoV-2-specific antibodies were not determined in convalescent plasma donors [28].

In contrast to SARS and MERS, where antibodies are detected for 2–3 years after infection [29,30], the duration of antibody titers in COVID-19 patients is still controversial. However, recent studies show that spike-specific antibodies are detected in patients after six months of recovery [31,32]. In addition, although antibodies against different viral epitopes including spike (S), nucleoprotein (N), or membrane protein (M) are detected, only those specific for spike may present therapeutic activity. Spike contains a receptor-binding domain (RBD) that is the responsible for interacting with the membrane receptor ACE2 allowing viral entry and cell infection. Thus, antibodies against RBD would present neutralizing activity, since they would block spike-ACE2 interaction. RBD is a region that seems to contain immunodominant epitopes against which antibodies are generated in COVID-19 patients [33]. In addition, other spike regions like the N-terminal domain have been shown to regulate its interaction with ACE2 and viral entry, thus also potentially being a target for neutralizing antibodies [34]. Thus, the validation of fast and robust methods to quantify the presence of neutralizing antibodies in plasma from COVID-19 convalescent patients is required in order to create plasma biobanks at hospitals in order to ensure the greatest efficiency and maximum performance of passive immunization in COVID-19.

Here, we have validated a previously developed in-house RBD IgG ELISA test [35] using a large cohort (more than 320 samples in total) of convalescent plasma and serum samples and adapted it to quantify the concentration of plasma RBD IgG and its correlation with the SARS-CoV-2 neutralizing activity in vitro.

## 2. Materials and Methods

### 2.1. Expression and Purification of RBD

The optimized DNA sequence encoding amino acid residues 319–541 of the Receptor-Binding Domain (*RBD*) was synthesized by Gen-Script (USA) for expression in HEK293 cells. The DNA, containing at the 5′-end a recognition sequence for KpnI, and at the 3′-end a stop codon and a recognition sequence for XhoI, was cloned into a modified pHLSec containing after the secretion signal sequence a 12xHis tag, a superfolder Green Fluorescent Protein (GFP) [36], and a tobacco etch virus (TEV) cleavage site, rendering the vector pHLSec-12His-GFP-TEV-RBD. Both the synthesis of the *RBD* construct and the engineered pHLSec together with the cloning of *RBD* into pHLSec-12His-GFP-TEV were performed by GenScript. pHLSec-12His-GFP-TEV-*RBD* was transfected into an HEK293F cell line (Thermo Fisher Scientific) as described below. Cells were grown in suspension in a humidified 37 °C and 8% CO_2_ incubator with rotation at 125 rpm. Transfection was performed at a cell density of 2.5 × 10^6^ cell/mL in fresh F17 serum-free media with 2% Glutamax and 0.1% P188. For each 150 mL of culture, 450 μg of the plasmid (1 μg/μL) was diluted to 135 μL with sterilized 1.5 M NaCl. This mixture was added to each 150 mL cell culture flask and incubated for 5 min in the incubator. After that, 1.35 mg of PEI-MAX (1 mg/mL) was mixed to 135 μL with sterilized 1.5 M NaCl and added to the cell culture flask. Cells were diluted 1:1 with pre-warmed media supplemented with valproic acid 24 h post-transfection to a final concentration of 2.2 mM. Cells were harvested 6 days post-transfection by spinning down at 300× *g* for 5 min, after which the supernatants were collected and centrifuged at 4000× *g* for 15 min. Supernatant was dialyzed against buffer A (25 mM TRIS pH 7.5, 300 mM NaCl) and loaded into a His-Trap Column (GE Healthcare). Protein was eluted with an imidazol gradient in buffer A from 10 mM up to 500 mM. Buffer exchange to 25 mM TRIS pH 7.5, 150 mM NaCl (buffer B) was carried out using a HiPrep 26/10 Desalting Column (GE Healthcare). TEV protease was then added in a ratio 1:50 (TEV:RBD) to the fusion construct in order to cleavage the His-GFP. After 20 h of reaction at 18 °C, the cleavage was satisfactorily verified through SDS-PAGE. TEV protease and GFP were removed from the solution using a His-Trap Column (GE Healthcare), and the RBD was collected from the flow-through. Quantification of protein was carried out by absorbance at 280 nm using the theoretical extinction coefficient, ε_280 nm_(RBD) = 33,350 M^−1^cm^−1^.

### 2.2. Human Samples

A total of 177 plasma samples were obtained from convalescent COVID-19 donors from the Biobank of the Aragon Health System. Patients qualified for the study based on the following criteria: (a) they were eligible for blood donation; (b) aged 18–55 years; (c) they had received a COVID-19 diagnosis by a test to detect viral RNA (RT-qPCR) or anti-SARS-CoV-2 antibodies; (d) they had one negative COVID-19 nasopharyngeal swab tests based on RT-qPCR; (e) they had been discharged from the hospital or did not present symptoms for more than two weeks; and (f) they did not present COVID-19 symptoms at time of or prior to the donation of convalescent plasma. As an independent cohort, 168 human serum samples from convalescent COVID-19 health care professionals from the Hospital Clinico Universitario Lozano Blesa were used. All samples were stored at −80 °C until used for ELISA tests. A total of 40 human plasma and 30 human serum samples from healthy donors collected before 2018 were used as the negative control (pre-COVID-19 samples).

Samples and data from patients included in this study were provided by the Biobank of the Aragon Health System (PT17/0015/0039), integrated in the Spanish National Biobanks Network, and they were processed following standard operating procedures with the appropriate approval of the relevant ethics and scientific committees.

### 2.3. Determination of Anti-RBD IgG Levels by ELISA

An in-house indirect ELISA for the detection of IgG specific for RBD was established. Ninety-six-well plates were coated overnight, at 4 °C with 100 ng RBD protein in PBS. Subsequently, the coating solution was removed, and the plate was washed three times with 200 μL per well of PBS + TWEEN 0.05% (wash buffer). Non-fat milk (3%) dissolved in PBS + TWEEN 0.05% was used as blocking solution. The plate was incubated with a blocking solution for 2 h at 37 °C. Serum or plasma samples were prepared in 1% non-fat milk dissolved in PBS + TWEEN 0.05% (buffer assay). The blocking solution was removed and 100 µL of serum or plasma samples were added per well. Samples were incubated for 1 h at 37 °C. All samples were run in duplicate. Next, the plates were washed five times with 200 μL of wash buffer and 100 μL of goat anti-human IgG-Fc horseradish peroxidase (HRP) conjugate (1:300.000; Thermo Fisher Scientific) was added. After 1 h, plates were washed seven times with the wash buffer and 100 μL of TMB (3,3′,5,5′-tetramethylbenzidine; Sigma–Aldrich, St. Louis, MO, USA) solution was added to each well. The substrate was incubated for 10 min and the reaction was stopped by the addition of 50 μL of 2 M sulfuric acid. The optical density at 450 nm (OD450) was measured using a Multi-Mode Microplate Reader (Synergy™ HT, BioTek, Winooski, VT, USA).

Background value was determined at OD450 in wells without RBDs. The corrected OD450 values were calculated by subtracting the background value from the OD values in RBD-coated wells.

In some cases, the concentration of IgG (mg/L) in serum or plasma samples was quantified by regression analysis using a standard, serially diluted recombinant human purified IgG (Thermo Fisher Scientific, Waltham, MA, USA).

The limit of detection (LOD) was calculated using human negative plasma samples (collected before 2018) as mean + 3 × SD. LOD was used as the cutoff value to discriminate between positive and negative samples. Sensitivity and specificity were calculated using the 2 × 2 table method including the true positive, true negative, false positive, and false negative values using plasma samples collected before 2018 and plasma samples from convalescent donors.

Tests with the commercial ELISA assay (SARS-CoV-2 RBD IgG ELISA Kit; MyBioSource) were performed according to the manufacturer’s instructions. Briefly, 100 µL of samples and a blank control were added into their respective wells. Plates were covered and incubated at room temperature for 1 h. The content of the wells was discarded. Plates were washed 3 times with 300 µL of wash buffer. 100 µL of detection solution were added to each well. Plates were incubated at room temperature for 1 h. After that time, plates were washed four times with the wash buffer. Once completely dry, 100 μL of substrate solution were added to each well. This substrate was left on the plates for 15 min and then the reaction was stopped by the addition of 100 μL per well of stop solution. The optical density at 450 nm (OD450) was measured using a Multi-Mode Microplate Reader (Synergy™ HT, BioTek).

### 2.4. Virus Isolation and Expansion in Cell Culture

The epithelial cell line, Vero E6, from the kidney of a *Cercopithecus aethiops*, was kindly provided by Júlia Vergara from the Centro de Investigación en Sanidad Animal IRTA-CReSA (Barcelona, Spain). Vero E6 cells were cultured in Dulbecco’s Modified Eagle Medium (Sigma) supplemented with 10% fetal bovine serum (FBS) (Sigma), 2 mM Glutamax (Gibco), 100 U/mL penicillin (Sigma), 100 µg/mL streptomycin (Sigma), 0.25 µg/mL amphotericin B (Sigma), 1% non-essential amino acids (Gibco), and 25 mM HEPES (4-(2-hydroxyethyl)-1-piperazineethanesulfonic acid; Biowest), referred as complete medium in T75 flasks for expansion, and kept at 37 °C, in a 5% CO_2_ humidified incubator. Complete medium with 2% FBS, denominated growth medium, was used for titration and neutralization tests.

SARS-CoV-2 virus was isolated from a COVID-19 patient at the Hospital Clínico Lozano Blesa (Zaragoza, Spain). Virus identity was confirmed by real-time PCR, electron microscopy, and RNA sequencing and classified as B1.1 linage [37]. The virus was seeded and passaged in Vero E6 cells to establish high-titer batches that were used in the neutralization assays. Vero E6 cells were seeded in that T75 flasks at a density of 10^5^ cells/mL in complete medium and after 48–76 h, the sub-confluent cell monolayer was washed with phosphate buffered saline (PBS). Cells were then infected with 1–2 mL of growth medium containing the virus. After one hour of incubation at 37 °C (with gentle shaking every 10 min), 10 mL of growth medium were added. The flasks were observed daily and the virus was harvested 76 h later, when a cytopathic effect (CPE) of 80–90% was visible. The culture medium was centrifuged at 4 °C, 1000× *g* for 5 min to remove the cell debris. Then the virus was concentrated using Lenti-X Concentrator (Clontech Laboratories) by mixing 1 volume of Lenti-X Concentrator with 3 volumes of the clarified supernatant and mixing by gentle inversion. The mixture was incubated for 30 min at 4 °C and then centrifuged at 1500× *g* for 45 min at 4 °C. The supernatant was carefully removed and the pellet was resuspended in 1 mL of growth medium. Samples were aliquoted and stored at −80 °C.

### 2.5. SARS-CoV-2 Titration

The virus was titrated in serial 1 log dilutions to obtain the 50% tissue culture infectious dose (TCID50) per mL using VERO-E6 cultures in 96-well plates. 10^4^ cells/well were seeded the day before the titration and the neutralization assays. The supernatant was discarded and 100 µL of the virus serial dilutions in growth medium were added to the wells. The plates were observed daily for a total of 72–96 h for the presence of virus CPE using an inverted optical microscope. The 50% endpoint titers were calculated according to the Ramakrishnan simple formula based on eight replicates per point for titration [38] using the Ramakrishan newly proposed method formula: log_10_ 50% endpoint dilution = ((total number of wells with CPE/number of wells per dilution) + 0.5) × log dilution factor.

### 2.6. Micro-Neutralization Assay

Serum and plasma samples were heat-inactivated for 30 min at 56 °C and two-fold serial dilutions, starting from 1:10, were performed and mixed with an equal volume of SARS-CoV-2 diluted in growth medium. The serum/plasma-virus mixture was incubated for 1 h at 37 °C and 5% CO_2_, and afterwards 500 TCID50 of SARS-CoV-2 were added by duplicate to a 96-well plate containing a semi-confluent Vero E6 monolayer whose supernatant had been previously discarded. Positive and negative controls using the virus or serum/plasma alone, respectively, were used. Plates were incubated for 72–96 h at 37 °C and 5% CO_2_.

CPE was studied using an inverted optical microscope and a colorimetric read-out using crystal violet. The supernatant of each plate was carefully discarded and 100 µL of 4% formaldehyde were added. After 1 h at room temperature, the formaldehyde was discarded and 50 µL of 1% crystal violet in 20% methanol in water were added and incubated for 15 min at room temperature. Plates were washed twice in water and CPE was seen with the naked eye or with an inverted microscope by trained operators. The neutralization titer was calculated as the highest serum dilution that protected more than the 50% of cells from CPE, taking into account that the starting dilution ratio of the serum to the cells was 1:20.

## 3. Results and Discussion

**Figure 1:**
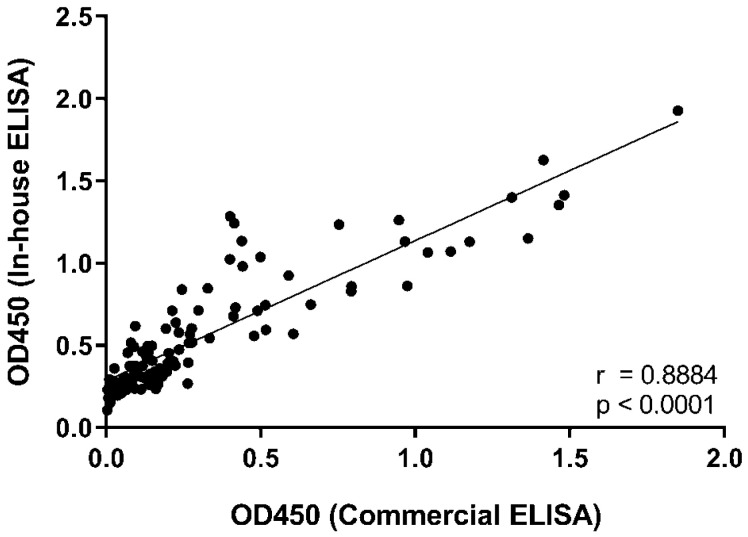
Correlation between a commercially available spike receptor-binding domain (RBD) IgG ELISA and the one developed in-house in convalescent plasma samples. The presence of RBD IgG was analyzed in plasma samples from convalescent COVID-19 patients (*n* = 177) by both a commercial ELISA and one developed in-house, as described in materials and methods. Absorbance values were represented and their correlation analyzed. Pearson r and P values (two-tailed) were determined using GraphPad Prism 8.0.2.

This study included 177 plasma donors that had recently recovered from COVID-19. The main characteristics of these donors are summarized in Table 1. COVID-19 was confirmed in all patients by at least a positive PCR test. The median age was 44 and approximately half of the patients corresponded to each gender. The median time from the onset of COVID-19 symptoms to donation was 44 days and 13.6% of the patients required hospitalization who were categorized as having had moderate/severe COVID-19 (Table 1). The rest of the clinical variables, such as fever or supplemental oxygen, are indicated in Table 1. In this cohort we aimed to validate in a large number of samples a recently developed indirect ELISA that employed recombinant RBD produced in mammalian cells as an immobilized antigen to quantify IgG against SARS-CoV-2 RBD in blood [35]. First of all, we compared the results obtained with this in-house developed ELISA (in-house RBD IgG ELISA) with those using a commercial ELISA (MyBiosource) that also detected RBD IgG. As shown in Figure 1, the correlation between both ELISA tests was very good confirming the efficacy of the in-house developed ELISA test to detect SARS-CoV-2 RBD-specific IgG.

Both the commercial and the previously described in-house ELISA tests [35] did not quantify antibody concentrations, which would be important step in providing a robust standardized protocol to optimize the use of plasma in passive immunization. Thus, we used the in-house ELISA to establish a quantitative RBD IgG test, in which convalescent plasma IgG concentration could be correlated with the plasma VN activity. To this aim, we established a calibration curve employing purified human IgG and the anti-human IgG secondary antibody employed as detection antibody in the in-house RBD IgG ELISA. Using this curve, the limit of detection of human IgG was established as 1 ng/mL. The absorbance values obtained in diluted plasma samples were extrapolated in the calibration curve and the concentration of RBD IgG was calculated. We determined the limit of detection of the RBD IgG ELISA test to establish a cutoff value in order to discriminate between positive and negative samples. To this aim, we used 20 plasma samples from pre-COVID-19 healthy donors that had been collected before 2018. The cutoff value was 80 ng/mL. A sample was considered as positive when the concentration of IgG was higher than the cutoff. Test sensitivity and specificity calculated using the pre-COVID-19 healthy donors and the convalescent plasma samples were 72% and 100%, respectively. The sensitivity value, although good, is relatively low, which is likely due to the samples from patients that have suffered from COVID-19 but have not generated IgG, which is known in COVID-19.

**Figure 2:**
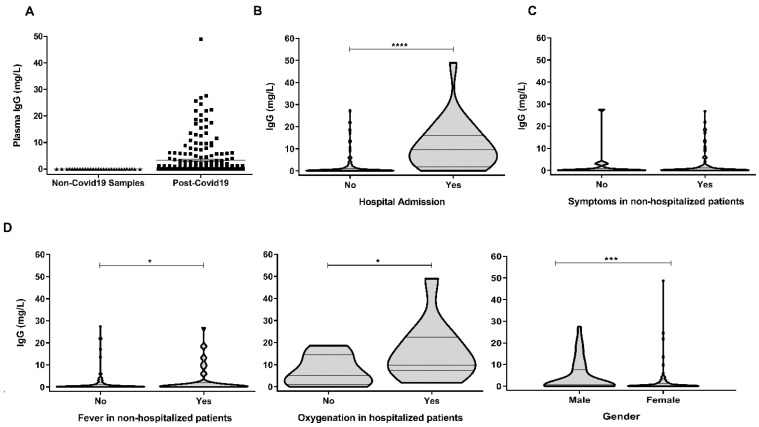
Quantification of RBD IgG in convalescent plasma samples and correlation with disease severity. (**A**) The concentration of RBD IgG in plasma samples from convalescent COVID-19 patients (*n* = 177) or from healthy donors before COVID-19 (*n* = 40) was calculated by indirect ELISA using a calibration curve prepared with known concentrations of purified human IgG diluted in plasma, as indicated in materials and methods. (**B**) The concentration of plasma RBD IgG was represented and compared between convalescent patients who did not require hospitalization and those that did. **** *p* < 0.0001 analyzed by the two-tailed Mann–Whitney test using GraphPad Prism 8.0.2. (**C**) The concentration of plasma RBD IgG was represented and compared between non-hospitalized symptomatic (*n* = 131) and asymptomatic (*n* = 22) patients. (**D**) The concentration of plasma RBD IgG was represented and compared between non-hospitalized afebrile (*n* = 106) and febrile (*n* = 47) patients, between hospitalized patients that required (*n* = 11) or not (*n* = 13) supplemental oxygen, and between male and female patients. * *p* < 0.05, *** *p* < 0.005 analyzed by the two-tailed Mann-Whitney test using GraphPad Prism 8.0.2.

As shown in Figure 2A, all plasma samples from pre-COVID-19 healthy donors that had been collected before 2018 tested negative for RBD IgG. Although the sample size is relatively small (40) this result suggests that the RBD IgG ELISA test specifically detects IgG against SARS-CoV-2 RBD, supporting a previous independent study employing 50 healthy donors [35]. In contrast, 72% of plasma samples from COVID-19 convalescent patients were positive for RBD IgG (Figure 2A). The minimum and maximum concentrations detected in positive samples were 0.087 and 45 mg/L, respectively, and the mean concentration was 3.3 mg/L. However, as can be seen in Figure 2A, there was a great variability between the concentrations detected in the different samples, with a standard deviation of six and a coefficient of variation of 200%.

Since more than half of convalescent plasma donors presented relatively low concentrations of RBD IgG in plasma (less than 1 mg/L), which may impact the efficacy of passive immunization, we decided to analyze if disease severity, classified according to the requirement for hospitalization, correlated with the concentration of plasma RBD IgG. As shown in Figure 2B, the concentration of RBD IgG was significantly higher in the convalescent patients who had required hospitalization than in those that were asymptomatic or presented mild symptoms who did not require hospitalization. In addition, in non-hospitalized patients, there were no differences between asymptomatic patients and those that presented mild symptoms (Figure 2C). In contrast, non-hospitalized patients with fever presented a significantly higher concentration of RBD IgG (Figure 2D). There were not significant differences between patients that required admission to the intensive care unit and those that did not (not shown), but hospitalized patients that required supplemental oxygen presented higher levels of RBD IgG (Figure 2D). In addition, male patients presented significantly higher levels of RBD IgG than female patients (Figure 2D).

These results indicate that the concentration of RBD IgG antibodies is increased in patients that had recovered from COVID-19 and required hospitalization and also in male patients and, thus, the application of this criterion might help to optimize donor selection and to improve the efficiency of passive immunization protocols for COVID-19. This finding is in line with previous studies indicating than moderate/severe cases of COVID-19 present higher levels of anti-SARS-CoV-2 IgG antibodies, albeit, in contrast to our study, most analyses were performed in hospitalized COVID-19 patients with active infection [39,40]. Similarly to our results, recent studies employing fewer donors have found that the presence of RBD IgG was higher in convalescent plasma from patients that had presented persistent and/or high fever [41] or that had required hospitalization [42]. Thus, our study employing a greater number of convalescent plasma donors validates these previous findings and, in addition, provides a novel quantitative test that might help to standardize donor selection.

**Figure 3:**
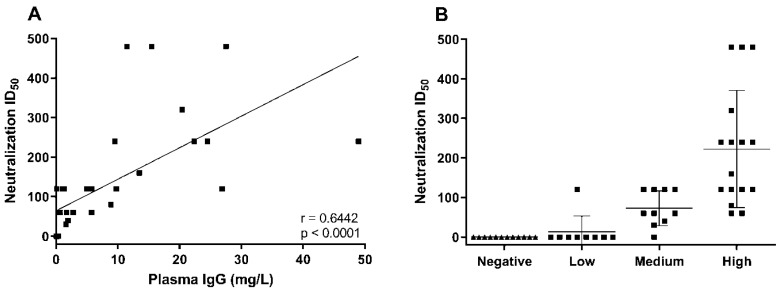
Correlation between plasma RBD IgG concentration and viral neutralizing (VN) activity. Plasma samples from convalescent COVID-19 patients (*n* = 35) were diluted two-fold, incubated with SARS-CoV-2 for viral neutralization, and the mixture added to a semi-confluent Vero E6 monolayer for cytopathic effect (CPE) determination. The neutralization EC50 was calculated as the highest dilution that protected more than 50% of the wells from CPE. (**A**) Correlation analysis between the concentration of plasma RBD IgG (mg/L) determined by ELISA and neutralization EC50 of plasma samples from convalescent COVID-19 patients (*n* = 35). Pearson r and P values (two-tailed) were determined using GraphPad Prism 8.0.2. (**B**) Samples were stratified into three groups depending on their RBD IgG concentration values (low > 0.3, 0.3 < medium < 2, high > 2 mg/L) calculated in Figure 2. Plasma samples from healthy donors taken pre-COVID-19 were used as control (*n* = 12).

Next, we analyzed if the concentration of RBD IgG correlated with the VN activity of plasma. To this aim, we selected plasma samples that contained different RBD IgG concentrations so that we could establish a concentration of IgG that was able to predict the VN of the plasma. VN activity was calculated as the plasma dilution that inactivated 50% of SARS-CoV-2 virus in cultures of Vero E6 cells, the so-called VN EC50 titer [38,43]. As shown in Figure 3A, the plasma concentration of RBD IgG correlated very well with plasma VN activity. It was found that most plasma samples that contained more than 3 mg/L RBD IgG presented a VN EC50 titer higher than 1:100, meaning that a plasma dilution of 1:100 inactivated 50% of the virus. We also calculated the plasma RBD IgG concentrations that presented a VN EC50 of 1:80 and 1:160, the levels recommended by FDA for the use of convalescent plasma for COVID-19 passive immunization, by using linear regression analyses in Figure 3A, which were found to be 1.96 and 12 mg/L, respectively. Based on these results samples were classified according to the RBD concentration as low (less than 0.3 mg/L), medium (between 0.3 and 2 mg/L), and high (more than 2 mg/L), finding that they grouped very well according to their VN activity (Figure 3B). Samples from pre-COVID-19 healthy donors did not show any VN activity confirming that the presence of antiviral activity in plasma from COVID-19 convalescent donors is specific for SARS-CoV-2-infected individuals.

**Figure 4:**
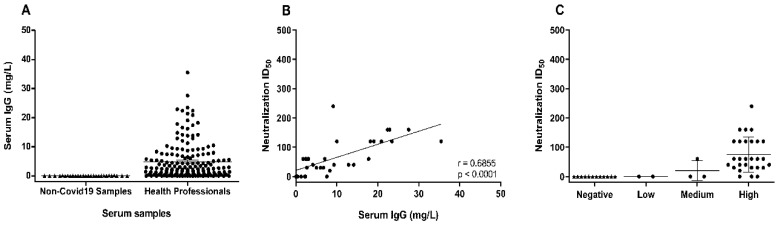
Quantification of RBD IgG in convalescent serum samples and correlation with VN activity. (**A**) The concentration of RBD IgG in serum samples from convalescent health workers (*n* = 168) or from healthy donors before COVID-19 (*n* = 30) was calculated by indirect ELISA using a calibration curve prepared with known concentrations of purified human IgG diluted in serum as indicated in materials and methods. (**B**) Serum samples from health professionals (*n* = 33) were diluted two-fold, incubated with SARS-CoV-2 for viral neutralization, and the mixture added to a semi-confluent Vero E6 monolayer for CPE determination. The neutralization ID50 was calculated as the highest dilution that protected more than 50% of the wells from CPE. Correlation analysis of the concentration of serum RBD IgG (mg/L) determined by ELISA and neutralization ID50 of serum samples from health workers (*n* = 33) was undertaken. Pearson r and P values (two-tailed) were determined using GraphPad Prism 8.0.2. (**C**) Samples were stratified into three groups depending on their IgG concentration values (low > 0.3, 0.3 < medium < 2, high > 2 mg/L). Serum samples from healthy donors before COVID-19 were used as control (*n* = 12).

Finally, we confirmed and expanded our results to other types of samples employing a different patient cohort and using serum instead of plasma. Here, prospectively collected serum samples from COVID-19 convalescent workers at the Hospital Clinico Lozano Blesa were selected and the concentration of RBD IgG was calculated. As shown in Figure 4A, serum from healthy donors collected before the emergence of COVID-19 did not present RBD IgG, confirming the results found in plasma samples. In contrast, most COVID-19 convalescent serum samples (93%) were positive for RBD IgG with concentration values ranging from 0.01 to 19 mg/L. The mean value of serum RBD IgG was 4.8 mg/L with a standard deviation of 6.3 and a coefficient of variation of 129%. These results were similar to those found in plasma samples, albeit they are not directly comparable since a donor cohort was used. Like in plasma samples, serum samples grouped very well according to the level of RBD IgG (Figure 4B) (where levels of less than 0.3, between 0.3 and 2, and more than 2 mg/L classed as low, medium, and high respectively), and thus, the correlation of serum RBD IgG concentration with VN EC50 titer was also very good (Figure 4C). It should be noted that the VN activity of serum was in general lower than in plasma samples. Serum samples with similar RBD IgG concentrations to plasma samples presented lower values of VN EC50 titer in serum than in plasma. The VN EC50 titer of 1:80 and 1:160 corresponded to 13.3 and 31.2 mg/L of RBD IgG in serum, respectively. Although these values are not directly comparable with plasma since they do not correspond to the same donors, they suggest that the VN activity of serum is lower than that of plasma, even if the same concentration of RBD IgG is present. This result might suggest that additional factors in plasma potentiate IgG VN activity. Alternatively, it could be that the different procedures used to collect the plasma or serum may have influenced these results [44]. Anyhow, the use of serum samples was aimed to confirm the utility of the ELISA test to quantify SARS-CoV-2 RBD IgG in convalescent COVID-19 patients. Thus, the correlation with serum VN activity is scientifically interesting albeit its clinical significance is limited since due to the manufacturing process plasma is mostly used for passive immunization protocols.

Similar to our analyses, other studies have found a good correlation between anti-spike antibody titers and VN activity determined by other methods, although the number of patients included in those studies was smaller than in ours [25,42,45,46].

Importantly, our study presents two clear differences with all previous ones. First we have established a quantitative RBD IgG ELISA test validated in two independent large cohorts of patients, and second we have determined the RBD IgG concentration that predicts the VN activity of convalescent plasma in vitro. The presence of high concentrations of RBD IgG, and thus, high VN activity, depends on the severity of COVID-19, as determined by hospitalization requirement and it is higher in male than in female patients. The main limitation of our study is the variability in the number of days since the onset of COVID-19 symptoms and plasma donation. In addition, it is not clear if our ELISA test that has been developed using RBD antigens from the first viral strain isolated in China in 2019 will efficiently detect IgG and predict the VN activity in samples from COVID-19 convalescent patients infected with new viral strains that present mutations in the RBD sequence, such as the B1.1.7 (N501Y RBD), B1.1351 (N501Y, K417S, E484K RBD), and P1 (N501Y, K417T, E484K RBD) variants. Based on the most recent evidences it has been found that the N501Y mutation only marginally affected the VN activity of plasma/serum from COVID-19 patients or from patients immunized with the original spike mRNA vaccines [47,48,49]. In contrast, the E484K mutation seems to significantly affect the VN activity of convalescent plasma or plasma from immunized individuals [47,48,50,51]. Notably, the detection of RBD-specific antibodies in plasma from convalescent patients was not affected if E484K RBD or wild-type RBD antigens [52] were used, suggesting that non-neutralizing RBD-specific antibodies are detected by ELISA. Thus, our ELISA and others based on wild-type RBD antigens might be able to detect mutated RBD IgG, albeit they might not be able to predict the VN activity in the plasma/serum from individuals infected with the P1 or B1.1351 variants containing the E484K mutation. Pending further study confirming the impact of different mutations on the VN activity of convalescent plasma from individuals infected with different variants, it might be advisable to develop ELISA tests in which different RBD sequences are used as antigens.

Preliminary clinical trials indicate that the selection of convalescent plasma donors with a high level of VN antibodies is key for therapeutic success [20,23,25,28,53,54]. Thus, pending of validation in future clinical trials and with the limitations described above, our results indicate that the establishment of the SARS-CoV-2 RBD IgG concentration in plasma samples would be very useful for optimal donor selection and effective passive immunization in COVID-19.

## 4. Conclusions

The concentration of RBD-specific IgG predicts the viral neutralizing activity of convalescent plasma and serum against SARS-CoV-2. Quantitative anti-RBD IgG detection systems might help to standardize and predict the efficacy of convalescent plasma against SARS-CoV-2 and to optimize the creation of COVID-19 convalescent plasma biobanks to treat the maximum number of patients with the highest efficacy.

## Figures and Tables

**Table 1:** Characteristics of convalescent plasma donors.

Age (Median; Range)	44 (20–64)
Gender (female)	92/177 (52%)
Asymptomatic	22/177 (12%)
Time from symptom onset (median; range)	45 (22–111)
Moderate/severe COVID-19 (hospitalization)	24/177 (13.6%)
Fever, non-hospitalized	47/153 (30.7%)
Oxygenation, hospitalized	11/24 (45.8%)

## Data Availability

The data presented in this study are available in article.
